# The Potential Value of m6A RNA Methylation in the Development of Cancers Focus on Malignant Glioma

**DOI:** 10.3389/fimmu.2022.917153

**Published:** 2022-05-30

**Authors:** Fan Chen, Xuan Xie, Min Chao, Haiyan Cao, Liang Wang

**Affiliations:** ^1^ Department of Neurosurgery, Tangdu Hospital of Fourth Military Medical University, Xi’an, China; ^2^ Reproductive Medicine Center, Department of Gynecology & Obstetrics, Xijing Hospital of Fourth Military Medical University, Xi’an, China

**Keywords:** m6A, cancer progression, GBM, immunotherapy, stem cells

## Abstract

N6-methyladenosine (m6A) RNA methylation is an epigenetic modification that has emerged in the last few years and has received increasing attention as the most abundant internal RNA modification in eukaryotic cells. m6A modifications affect multiple aspects of RNA metabolism, and m6A methylation has been shown to play a critical role in the progression of multiple cancers through a variety of mechanisms. This review summarizes the mechanisms by which m6A RNA methylation induced peripheral cancer cell progression and its potential role in the infiltration of immune cell of the glioblastoma microenvironment and novel immunotherapy. Assessing the pattern of m6A modification in glioblastoma will contribute to improving our understanding of microenvironmental infiltration and novel immunotherapies, and help in developing immunotherapeutic strategies.

## Introduction

The m6A modification has been recognized as one of the markers of post-transcriptional regulation in diverse types of RNAs, such as transfer RNA, circular RNA, long non-coding RNA, messenger RNA, ribosomal RNA and microRNA (1, 2). RNA m6A modifications have been shown to play a significant role in regulating RNA translation, splicing, translocation, stability, and higher structure (3, 4). Almost all types of RNA have been found to contain m6A modifications to date (5, 6). In humans, there are over 7000 genes with 12,000 m6A sites enriched in the consensus sequence RRACH (H=A, C or U, R=G or A), which tend to occur in the stop codon and 3′ untranslated regions (3′ UTRs) (3, 7). The dynamic regulation of m6A modifications is primarily dependent on m6A methyltransferases/writers, which can be cleared by demethylases/erases and recognized by m6A-binding proteins/readers (8, 9). Recently, an increasing number of studies have demonstrated that m6A plays an essential role in the occurrence and progression of cancer (10, 11).

Glioblastoma (GBM) is the most common and most malignant tumor of the adult central nervous system, which accounts for roughly half of all primary brain tumors and practically 60% of all types of gliomas (12, 13). Despite the use of comprehensive therapies such as surgery, radiotherapy, and novel immunotherapies, the 2-year survival rate for GBM is only 15 months, and reliable biomarkers and effective immunotherapy targets for GBM are still lacking (6, 14). Patients with GBM present a complex state of immune dysfunction involving several mechanisms of immunosuppression and tolerance, and immunotherapy has emerged as a novel approach to GBM treatment (6, 15). Studies have demonstrated that GBM is more heterogeneous than peripheral tumors, which indicates that several factors including RNA modification, tumor microenvironment (TME), and stem cell phenotype may influence immune checkpoint blockade therapy and developmental plasticity in GBM (16). m6A modifications influence the formation of multiple TMEs, including GBM, and are involved in cancer stem cell (CSC) generation and maintenance, and immunotherapy resistance, making the investigation of m6A methylation offers a new perspective for the treatment of GBM (17, 18).

The novel immune checkpoint blockade therapies (anti-PD-1 and PD-L1) are now showing satisfactory efficacy in some cancer patients. The m6A modifications are closely related to novel immune checkpoint blockade therapies. As an m6A demethylase, FTO can induce resistance to anti-PD-1 treatment in melanoma cells (19). It has been shown that both METTL3 and METTL14 contribute to the development and tumorigenesis of human glioma stem cells (GSCs), and METTL3 overexpression or suppression of FTO inhibits GSC self-renewal and growth (20). Our previous study also observed an association between anti-PD-L1/PD-1 treatment response and m6A modification patterns, confirming that m6A modification patterns in GBM affect the infiltration of immune cells in the GBM microenvironment (6).

In this review, we analyzed the correlation between m6A modifications, m6A regulators and GBM as well as peripheral cancers. We clarified the correlation of m6A modification in the occurrence and development of GBM as well as peripheral cancers, analyzed the molecular, immune cell infiltration in TME, and stemness characteristics of GBM cells with distinct m6A modification patterns, and the influence of m6A modification patterns on novel immunotherapies for GBM as well as peripheral cancers.

## Overview of m6A

**Figure 1:**
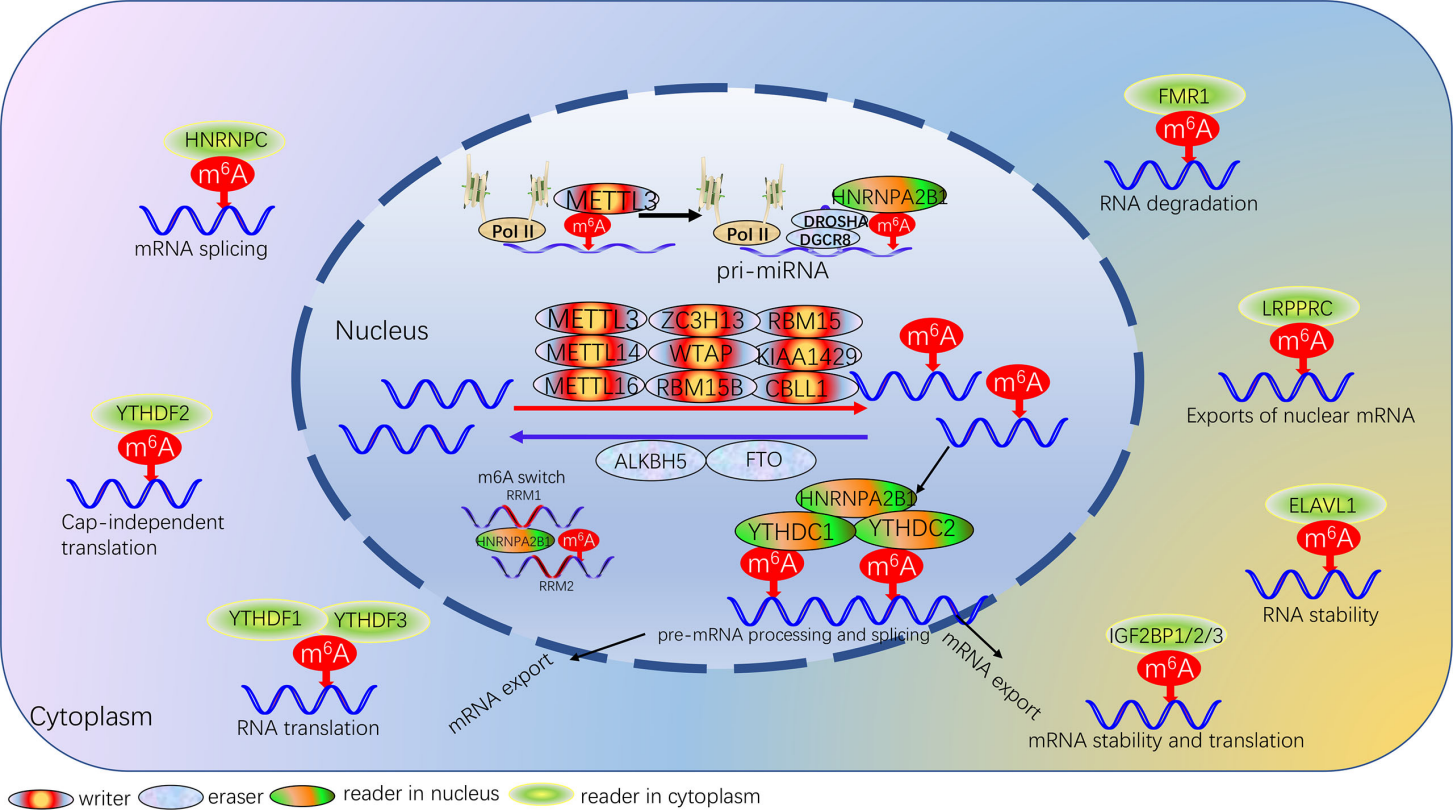
Landscape of dynamic and reversible processes of m6A RNA methylation mediated by 24 regulators and their potential biological functions for RNA.

This is a dynamic and reversible biological process that m6A is formed by the m6A methyltransferases complex. The function of m6A modification is achieved by RNA methyltransferases (writers: METTL3, METTL14, METTL16, RBM15, RBM15B, WTAP, KIAA1429, ZC3H13, CBLL1) (21–28), RNA demethylases (erasers: FTO, ALKBH5) (29, 30) and m6A binding proteins (readers: YTHDC1, YTHDC2, HNRNPC, HNRNPA2B1, YTHDF1, YTHDF2, YTHDF3, IGF2BP1, IGF2BP2, IGF2BP3, FMR1, LRPPRC, ELAVL1) (31–41), which can recognize, remove or add m6A modification sites and modify essential biological processes accordingly. m6A modification processes occur mainly in adenines of the RRACH sequence (42). We summarized the protein types involved in m6A modifications and described the biological functions of each protein (**Table 1**; **Figure 1**).

**Table 1:** 24 m6A regulators and their functional roles in RNA metabolism.

Type	Regulator	Location and Funtion	References
Writer	METTL3	Mediates m6A modifications	21
	METTL14	Assists METTL3 to catalyze m6A RNA methylation	22
	METTL16	Mediates m6A modifications	23
	WTAP	Promotes formation of the METTL3-METTL14 m6A methyltransferase complex	24
	KIAA1429	Directs methyltransferase components to specific RNA regions	25
	RBM15	By binding the m6A complex and recruiting it to specific RNA site	26
	RBM15B		
	ZC3H13	Bridges WTAP to the mRNA-binding factor Nito Mediates m6A modifications	27
	CBLL1		28
Eraser	FTO	Demethylates m6A modifications	29
	ALKBH5	Demethylates m6A modifications	30
Readers	YTHDC1	Promotion of RNA translocation and splicing	31
	YTHDC2	Enhances the translation of target RNA and expedites mRNA decay	32
	HNRNPC	Mediates mRNA splicing	33
	HNRNPA2B1	Promotes primary miRNA processing	34
	YTHDF1	Promotes RNA translation initiation by binding to initiation factors	35
	YTHDF2	Destabilization of mRNA	36
	YTHDF3	Promotes translational efficiencies	37
	IGF2BP1	Enhances mRNA stability and translation	38
	IGF2BP2		
	IGF2BP3		
	FMR1	Contributes to maternal RNA degradation	39
	LRPPRC	Exports of nuclear mRNA	40
	ELAVL1	Mediates the RNA stability	41

### RNA Methyltransferases/Writer

The multicomponent methyltransferase complex is involved in catalyzing the formation of m6A modifications (43). The multicomponent methyltransferase complex consists mainly of METTL3/METTL14 heterodimers and a variety of other methyltransferases (43). METTL3 is an S-adenosylmethionine-binding protein, the core enzyme that exerts methyltransferase activity in the methyltransferase complex, and is the first characteristic component of the m6A methyltransferase complex (8). METTL14 is the second supporting enzyme, and the two co-localize in the nuclear speckle and form a stable heterocomplex in a 1:1 ratio (44). METTL3 acts primarily as the catalytic core, while METTL14 is the structural carrier for RNA binding, where the C-terminal arginine-glycine repeat sequence is the secondary RNA substrate binding site and is essential for METTL3-METTL14 catalytic activity (45). METTL16 is a homolog of METTL3, which deposits m6A into hundreds of specific messenger RNA targets in the nucleus; in the cytoplasm, METTL16 contributes to translation in an m6A-independent manner (46). WTAP has no conserved catalytic methylation structural domain, but WTAP can interact with METTL3 and METTL14 as an adaptor protein, thereby significantly affecting cellular RNA m6A methylation (45). KIAA1429, RBM15 and its homologs RBM15B and ZC3H13 are components of the m6A methyltransferase complex and are essential for m6A methylation. The KIAA1429 knockout resulted in a 4-fold decrease in the m6A peak score (47). RBM15 and RBM15B modulated m6A modification by binding target RNAs and recruiting methyltransferase complexes (26). ZC3H13 plays a key role in anchoring Virilizer, WTAP and Hakai in the nucleus to promote m6A methylation (27). CBLL1 regulates selective splicing and promotes exon skipping and intron retention in selective splicing events (28).

### RNA Demethylases/Eraser

To date, a total of two m6A demethylases have been fully investigated, which are fat mass and obesity-associated protein (FTO) and α-ketoglutarate-dependent dioxygenase homolog 5 (ALKBH5). FTO is mainly located on chromosome 16q12.2, and FTO regulates m6A levels of downstream targets mainly through its 3’ untranslated region (48). Studies have shown that FTO is an important component of m6A modification, which not only plays a key role in obesity-related diseases but also participates in the occurrence, development, and prognosis of many cancers, regulates cancer stem cell function, self-renewal and metastasis (48). ALKBH5 plays a dual role in a variety of cancers by regulating various biological processes such as proliferation, invasion, migration and metastasis (49). The basic regulatory mechanism of ALKBH5, which relies on m6A-dependent modifications, is associated with long non-coding RNAs, CSCs, hypoxia and autophagy (50).

### m6A Binding Proteins/Reader

The reader protein of m6A can recognize and bind m6A-modified transcripts to regulate gene expression by regulating multiple processes, such as mRNA stability, structure, splicing, export, translation efficiency, and miRNA biogenesis (31, 51–54). The YT521-B homology (YTH) family, which functions as the major reading protein to recognize m6A-modified mRNAs and regulate target gene expression, consists of five proteins, including YTHDC1, YTHDC2, YTHDF1, YTHDF2 and YTHDF3, all of them with a conserved m6A binding domain and bind preferentially to the m6A-modified region RNA on the consensus sequence of Rm6ACH (55). The m6A reader also includes some members of the heterogeneous nuclear ribonucleoprotein (HNRNP) family. HNRNPA2B1 can recognize specific targets containing AGG and UAG motifs through the RRM1 and RRM2 structural domains, and can also directly regulate the processing of m6A-modified transcripts by interacting with DGCR8, a miRNA microprocessor complex protein (34, 56). HNRNPC regulates mRNA splicing and abundance by processing m6A-modified RNA transcripts, while m6A influences the secondary structure of RNA and promotes the binding of transcripts to HNRNPC to regulate mRNA splicing and abundance, a process also known as the “m6A switch” (57). Insulin-like growth factor 2 mRNA binding protein (IGF2BP) also recognizes m6A modifications and is another family of m6A readers, including IGF2BP1/2/3 (38). IGF2BP family in contrast to the mRNA decay-promoting features of YTHDF2 of the YTH family, IGF2BP1/2/3 promote the storage and stability of their target mRNAs in an m6A-dependent manner. FMR1 binds preferentially to mRNAs containing the m6A-tagged “AGACU” motif with high affinity, and this high-affinity binding is dependent on the hydrophobic network within the FMR1 KH2 structural domain (39). Other m6A readers include the leucine-rich pentatricopeptide-repeat containing (LRPPRC) (58) and ELAV Like RNA Binding Protein 1 (ELAVL1) (59).

## m6A Modification and Solid Tumors

**Figure 2:**
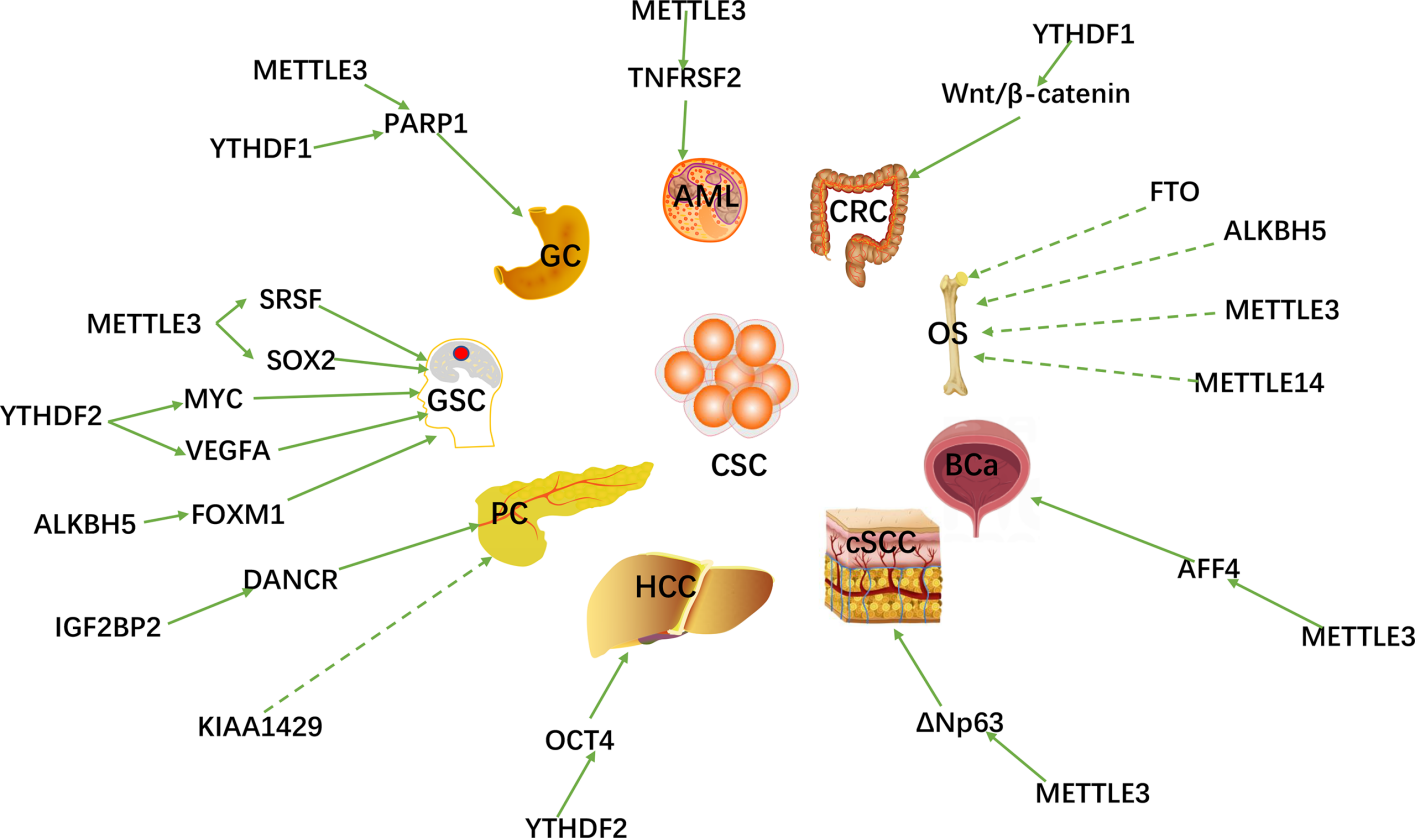
The m6A regulators are involved in various peripheral CSCs and GSCs.

**Figure 3:**
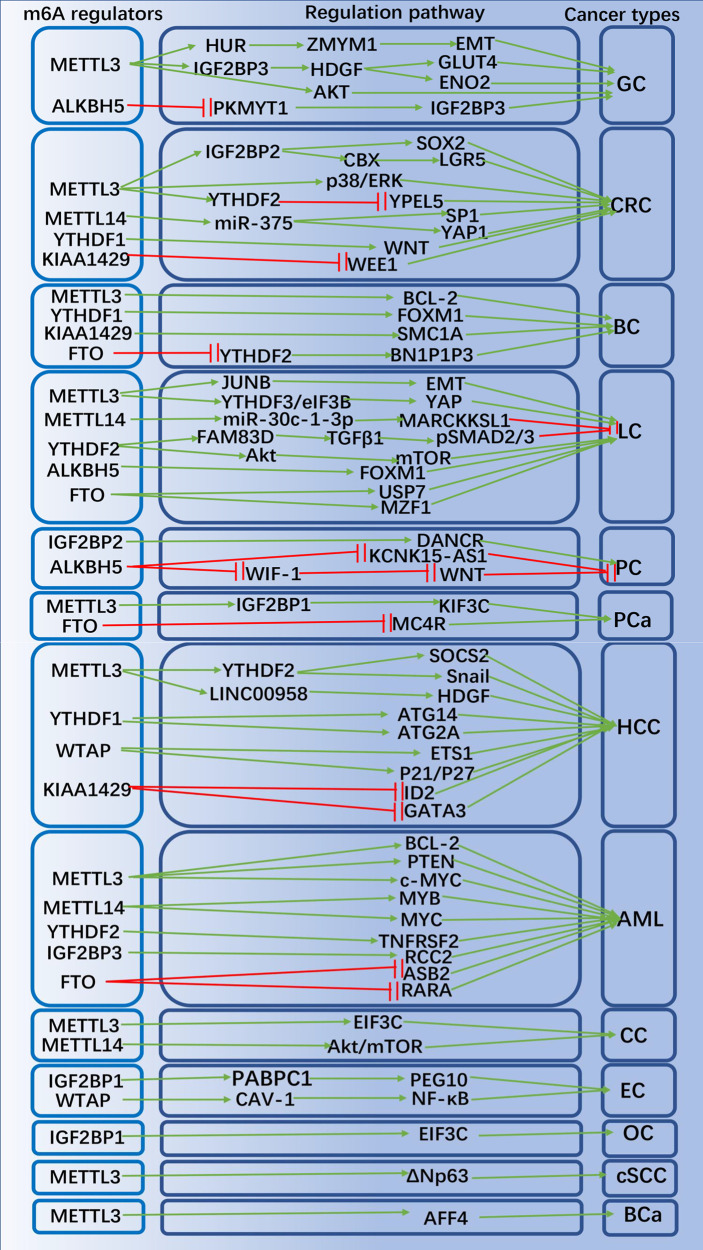
The potential role of m6A modification in peripheral cancers progression and related mechanisms. The m6A regulators promote or inhibit peripheral cancer progression by affecting the expression of tumor-associated genes.

With the breakthroughs in the identification and understanding of m6A writers, erasers and readers, m6A methylation has been shown to affect virtually every aspect of RNA metabolism, including RNA expression, translation, splicing, decay, nuclear export, and RNA-protein interactions (60, 61). Recently, there is growing evidence that writers, erasers and readers of m6A RNA modifications are related to multiple types of human cancers, including: gastric cancer (GC) (62), colorectal cancer (CRC) (63), breast cancer (BC) (64), lung cancer (LC) (65), hepatocellular carcinoma (HCC) (66), pancreatic cancer (PC) (67), prostate cancer (PCa) (68), acute myeloid leukemia (AML) (69), cervical cancer (CC) (70) ovarian (71) and endometrial cancer (72) (OC and EC), etc. m6A RNA methylation plays an important role in promoting CSC self-renewal, proliferation and resistance of cancer cell to radiation or chemotherapy. Here, we review and summarize the latest research on m6A methylation in various cancers (**Figure 2**) and CSCs (**Figure 3**).

### Gastric Cancer

GC is the world’s fifth most prevalent cancer and the third most mortality cancer (73). Studies of TCGA and CGGA databases have demonstrated that assessment of m6A modification patterns within GC can predict GC inflammatory stage, subtype, TME immune cell infiltration, genetic variation, and prognosis of patients (74). In GC subtypes with high m6A signaling, TME stromal activation and absence of effective immune infiltration were identified, suggesting a non-inflammatory as well as immune-exclusion phenotype of TME with poorer patient survival (74). Mechanistically, METTL3 expression is elevated in GC, and the m6A modification of zinc finger MYM-type containing 1 (ZMYM1) mRNA by METTL3 requires a HUR-dependent pathway to improve its stability, and ZMYM1 recruits the CTBP/LSD1/COREST complex to bind to the E-cadherin promoter and mediates E-calmodulin promoter inhibition, which in turn promotes the EMT program and migration of cells (75). Activation of H3K27 acetylation induced transcription of METTL3 and stimulated m6A modification of HDGF mRNA, and IGF2BP3 was subsequently recognized and bound directly to the m6A site on HDGF mRNA, enhancing the stability of HDGF mRNA (76). Secreted HDGF facilitated tumor angiogenesis, while nuclear HDGF stimulated ENO2 and GLUT4 expression, followed by increased GC cell glycolysis, promoting tumor growth and metastasis (76, 77). It has also been demonstrated that down-regulation of METTL3 expression in human GC cells inhibits the proliferation and migration of tumor cells and inactivates the signaling pathway of Akt (78, 79). ALKBH5 regulated PKMYT1’s expression in an m6A-dependent manner, and IGF2BP3 contributed to stabilizing the mRNA stability of PKMYT1 through its m6A modification site, which acted as a downstream target of ALKBH5 and facilitated GC migration and invasion (80). METTL3 promoted resistance to oxaliplatin in CD133+ GC stem cells by increasing the stability of PARP1 mRNA and enhancing the activity of the base excision repair pathway (81). Besides, m6A-associated lncRNA signatures can independently predict GC survival and correlate with immunotherapeutic response to GC (82).

### Colorectal Cancer

CRC is the third most prevalent of all malignancies and second in cancer-related mortality worldwide (83). CRC expresses high levels of METTL3, which is a marker of poor prognosis. IGF2BP2 recognized methylated SOX2 transcripts, especially the CDS region, thereby extending the half-life of SOX2 mRNA. SOX2 is a downstream gene of METTL3, and in CRC, SOX2 expression was positively associated with the expression of METTL3 and IGF2BP2. The oncogene METTL3 inhibits SOX2 degradation *via* IGF2BP2, thus increasing SOX2 expression (84). METTL3 could target the m6A site in the coding sequence region of the YPEL5 transcript and epigenetically repress YPEL5 in an m6A-YTHDF2-dependent manner to promote the growth and metastasis of colorectal cancer (85). METTL3 relies on IGF2BP1 to extend the half-life of chromo box 8 (CBX8) mRNA, which recruits Pol II and KMT2B to the promoter of leucine-rich repeat sequence of G protein-coupled receptor 5 (LGR5) and maintains the H3K4me3 state, ultimately maintaining CRC stemness and promoting its drug resistance (86). In CRC, METTL3 downregulation revealed to activate phosphorylation of p38 and ERK; METTL3 also inhibited proliferation, invasion and migration of CRC cells *via* the p38/ERK pathway (87). METTL14 inhibited the proliferation of CRC cells *via* the miR-375/YAP1 pathway and suppressed the invasion and migration of CRC cells *via* the miR-375/SP1 pathway (88). KIAA1429 exerted oncogenic effects in CRC cells by inhibiting the expression of WEE1 in an m6A non-dependent manner and was associated with low survival rates in CRC patients (89). Knockdown of YTHDF1 could significantly inhibit the WNT/β-linked protein pathway activity in CRC cells and suppressed the biological activity of CRC cells (90). YTHDF1 is critical for CRC stem cell-like activity and tumorigenesis in CRC (90).

### Breast Cancer

BC is the most frequently diagnosed cancer in women worldwide (91). METTL3 facilitated m6A modification of the 3′ UTR of B-cell/lymphoma 2 (BCL-2) mRNA, which in turn promoted cell proliferation, migration and suppressed apoptosis through upregulation of BCL-2 expression (92). YTHDF1 facilitated BC metastasis by recognizing and binding to the m6A-modified mRNA of FOXM1 and accelerating FOXM1’s translation process (93). KIAA1429 could bind to the motif in the SMC1A mRNA’s 3’UTR and strengthen the stability of SMC1A mRNA, promoting migration and invasion of BC cells (94). The eraser FTO was demonstrated to be highly expressed in BC tissues, and the pro-apoptotic gene BCL2-interacting protein 3 (BNIP3) is a downstream target of FTO-mediated m6A modification. FTO can mediate the demethylation of m6A in the 3′ UTR of BNIP3 mRNA and promote its degradation through a mechanism dependent on YTHDF2 (95).

### Lung Cancer

LC ranks first among the causes of cancer-related deaths in the world (96). METTL14 facilitated the maturation of miR-30c-1-3p and mediated the expression of its target gene MARCKSL1 through miR-30c-1-3p to suppress the progression of LC (97). YTHDF2 could regulate the activity of the FAM83D-TGFβ1-pSMAD2/3 pathway, which in turn inhibited the invasion and migration of LC cells (98). METTL3 promoted yes-associated protein (YAP) mRNA translation through YTHDF1/3 and eIF3B, and increased the stability of YAP mRNA by regulating the MALAT1/miR-1914-3p/YAP axis to induce non-small cell lung cancer (NSCLC) treatment metastasis and resistance b (99). METTL3 promoted m6A modification, enhanced total mRNA levels and strengthened mRNA stability of the EMT transcriptional regulator JUNB, playing an essential role in TGF-β-induced EMT in LC (100). FTO enhanced myeloid zinc finger 1 (MZF1) expression by decreasing the level of m6A in MZF1 mRNA and strengthening its stability, thereby promoting LC development (101). Upregulation of FTO expression reduced the m6A level of ubiquitin-specific peptidase 7 (USP7), increased the USP7 mRNA stability, and promoted the development of NSCLC (102). ALKBH5 reduced m6A modification of FOXM1 mRNA and promoted FOXM1 expression, thereby affecting the proliferation and migration of LC cells (103). Overexpression of hypoxia-mediated YTHDF2 promoted LC cells proliferation and migration by activating the AKT/mTOR axis, and overexpression of YTHDF2 induced the EMT process in LC (104).

### Hepatocellular Carcinoma

HCC is a malignant tumor with high incidence and poor prognosis,usually found in patients with chronic liver disease (105). Studies have shown that combined the levels of METTL3 and YTHDF1 could be used as a biological indicator to indicate the degree of malignancy and to assess the prognosis for patients with HCC (106). METTL16 functions as an m6A writer and a translation initiation facilitator, both of which together exert their facilitating roles in HCC genesis (46). YTHDF1 promoted the translation of autophagy-associated genes ATG14 and ATG2A through binding to m6A-modified ATG14 and ATG2A mRNAs, thereby promoting the progression of HCC (107). Analysis of the GO and KEGG pathways of genes co-expressed with YTHDF1 in HCC from the TCGA database showed that YTHDF1 plays an essential role in modulating the cell cycle and metabolism of HCC cells (108). METTL3 could promote the progression of HCC through the following mechanisms: METTL3 increased the degradation of SOCS2 mRNA *via* an m6A-YTHDF2-dependent manner, increased m6A expression in SOCS2 mRNA, and suppressed SOCS2 expression in HCC; METTL3-mediated m6A modification promoted LINC00958 expression *via* stabilizing LINC00958’s RNA transcripts, and consequently increased HDGF expression *via* spongy miR-3619-5p; METTL3 could facilitate translation of the key EMT transcription factor Snail by installing m6A in its coding sequence and 3′ UTR region, and interactions involving YTHDF1 and eEF-2 increased snail translation (109–111). KIAA1429 is highly expressed in HCC tissues, and KIAA1429 contributed to the migration and invasion of HCC cells by increasing the level of m6A in DNA binding inhibitor 2 (ID2) mRNA and inhibiting its expression (112). KIAA1429 caused RBP HUR segregation and GATA3 pre-mRNA degradation by inducing 3′UTR methylation of GATA-binding protein 3 (GATA3) pre-mRNA (47). The effect of WTAP on HCC progression is mainly through its m6A modification leading to post-transcriptional repression of ETS1, and another mechanism is that the p21/p27-dependent pathway can modulate the cell cycle of HCC cells (113). YTHDF2 exerted inhibitory effects on HCC cell proliferation and angiogenesis *via* mRNA for IL11 and serpin family E member 2 (SERPINE2) (114). Knockdown of YTHDF2 significantly suppressed the number of HCC stem cell spheres and reduced the number of CD133+ stem cells (115). Inhibition of YTHDF2 impaired m6A methylation of the OCT4 mRNA 5′-UTR, which is responsible for regulating HCC stem cells, leading to translation inhibition of OCT4 (115).

### Pancreatic Cancer

PC is a highly aggressive disease that is expected to be the second leading cancer-related cause of death worldwide by 2030, usually presenting as a locally advanced or metastatic disease with a lack of effective treatments (116). It was shown that knockdown of METTL3 enhances PC sensitivity to chemotherapeutic drugs but has little effect on cell proliferation. By analyzing PC samples in the database, METTL3 was correlated with ubiquitin-dependent processes, mitogen-activated protein kinase cascades, RNA splicing and modulation of cellular processes (117). Overexpression of ALKBH5 sensitized PC cells to anticancer drugs and inhibited PC progression by reducing m6A-dependent WNT inhibitory factor 1 (WIF-1) levels and hindering its activation (118). ALKBH5 downregulated the expression of KCNK15-AS1 in PC cells by demethylation of KCNK15-AS1 and ultimately inhibited KCNK15-AS1-mediated migration and invasion of PC cells (119). IGF2BP2 can act as a reader for the m6A-modified lncRNA DANCR and play a role in promoting DANCR stabilization, which in turn co-promotes the stem cell-like properties of cancer and PC pathogenesis (120). The study demonstrated that KIAA1429 is essential in maintaining the stemness properties of PC cells (121). IGF2BP2 could mediate long non-coding RNA DANCR stability and contribute to the self-renewal of PC stem cells (120).

### Cervical, Ovarian and Endometrial Cancer

CC, OC and EC are the three common malignant tumors in women worldwide and are the leading causes of cancer-related deaths in women (12, 122). METTL3 could promote the proliferation of CC cells by targeting the 3’-UTR of hexokinase 2 (HK2) mRNA, and METTL3 also could promote aerobic glycolysis of CC by recruiting YTHDF1 to enhance the stability of HK2 (70). When METTL14 was knocked out in CC cells, their cell cycle was arrested. METTL14 silencing inhibited signaling pathway of PI3K/Akt/mTOR by suppressing phosphorylation of Akt and mTOR, and the expression of downstream apoptosis-related proteins was also affected (123). YTHDF1 enhanced EIF3C translation in an m6A-dependent manner by binding to m6A-modified EIF3C mRNA, while promoting overall translational output, thereby promoting tumorigenesis and metastasis in OC (124). IGF2BP1 recognized the m6A site in the 3’UTR of paternally expressed gene 10 (PEG10) mRNA and recruited polyadenylate-binding protein 1 (PABPC1) to strengthen the stability of PEG10 mRNA and increase the expression of PEG10 protein, thereby promoting EC cell proliferation (125). Decreased m6A methylation leads to lower expression of AKT negative regulator PHLPP2, increased expression of AKT positive regulator mTORC2, mutations in METTL3 or METTL14 may lead to increased proliferation of EC cells through this pathway (126). It was confirmed that WTAP could promote EC progression by methylating the 3’-UTR of CAV-1 and down-regulating the expression level of CAV-1 to activate the signaling pathway of NF-κB in EC (127).

### Acute Myeloid Leukemia

AML is the most frequent form of acute leukemia in adults, with a very high mortality rate (128). m6A was demonstrated to facilitate the translation of BCL2, PTEN and c-MYC mRNA in human AML cells (129). METTL3 mRNA and protein expression were upregulated in AML cells, and deletion of METTL3 in AML cell lines induced differentiation and apoptosis in recipient mice and delayed the progression of AML (129). WTAP has also been shown to be upregulated in AML cells and to play an essential role in the abnormal proliferation and inhibition of differentiation of leukemic cells (130). METTL14 was highly expressed in AML cells carrying t(11q23), t (15, 17) or t(8;21) translocations and was downregulated during myeloid differentiation (131). Knockdown of METTL14 facilitated AML and normal HSPC cells’ myeloid terminal differentiation and suppressed the proliferation of AML cells. METTL14 could modulate its target mRNAs, such as MYB and MYC, by m6A modification, which was negatively modulated by SPI1, demonstrating the role of SPI1-METTL14-MYB/MYC signaling axis in hematopoiesis and AML cells (131). FTO was demonstrated to be highly expressed in AML with t(11q23)/MLL rearrangements or t(15;17)/PML-RARA, FLT3-ITD and/or NPM1 mutations, promoting AML progression (132). FTO promoted leukemia oncogene mediated cell transformation and leukemia by reducing m6A levels in mRNA transcripts, inhibited all-trans retinoic acid (ATRA) induced AML cell differentiation, and modulated the expression of its target genes such as retinoic acid receptor alpha (RARA) and ankyrin repeat and SOCS box containing 2 (ASB2) (132). It has also been demonstrated that IGF2BP3 is required to maintain the survival of AML cells in an m6A-dependent manner and that IGF2BP3 functions to promote AML progression by interacting with RCC2 mRNA and stabilizing the expression of m6A-modified RNA (133). Studies have shown that overexpression of YTHDF2 in AML cells causes decreased half-life of a wide range of m6A transcripts, including TNF receptor superfamily member 2 (TNFRSF2) transcripts, which could help maintain the function of leukemic stem cells, and enhanced hematopoietic stem cell activity when YTHDF2 is knocked down (134).

### Other Cancers

Previous studies confirmed that METTL3 promotes bladder cancer (BCa) progression by regulating AF4/FMR2 family member 4 (AFF4) after m6A-directed transcription (135). Moreover, METTL3 levels and RNA m6A abundance were significantly increased in BCa stem cells, and knockdown of METTL3 caused impaired aldehyde dehydrogenase activity and sphere formation ability, which effectively inhibited the self-renewal of BCa stem cells (136). METTL3 expression was observed to be significantly upregulated in cutaneous squamous cell carcinoma (cSCC) tissues, and knockdown of METTL3 led to a significant decrease in the level of the undifferentiated marker k14 and a significant increase in the early differentiation marker K10 in cSCC cells, significantly inhibiting the stem cell-like properties of cSCC cells (137). Knockdown of METTL3 gene in cSCC cells reduced m6A levels and ΔNp63 expression in cSCC and inhibited cSCC cell proliferation, which could be restored when exogenous ΔNp63 was added (137). Wang et al. demonstrated the interaction between m6A modification and osteosarcoma (OS) stem cells, with METTL14 and FTO expression showing a significant decrease in OS stem cells, and elevated METTL3 and ALKBH5 expression in OS stem cells are closely associated with relatively low metastasis-free survival (138). Li et al (139). demonstrated that FTO modulates the proliferation, invasion and migration of PCa by regulating the expression level of melanocortin 4 receptor (MC4R), and that high expression of FTO partially reversed the promotion of the malignant phenotype of PCa cells by high expression of MC4R. In PCa cells, METTL3 could induce m6A modification on KIF3C and promote the stabilization of KIF3C mRNA through IGF2BP1 to promote PCa growth, migration and invasion (140).

## m6A Modification and Glioblastoma

**Figure 4:**
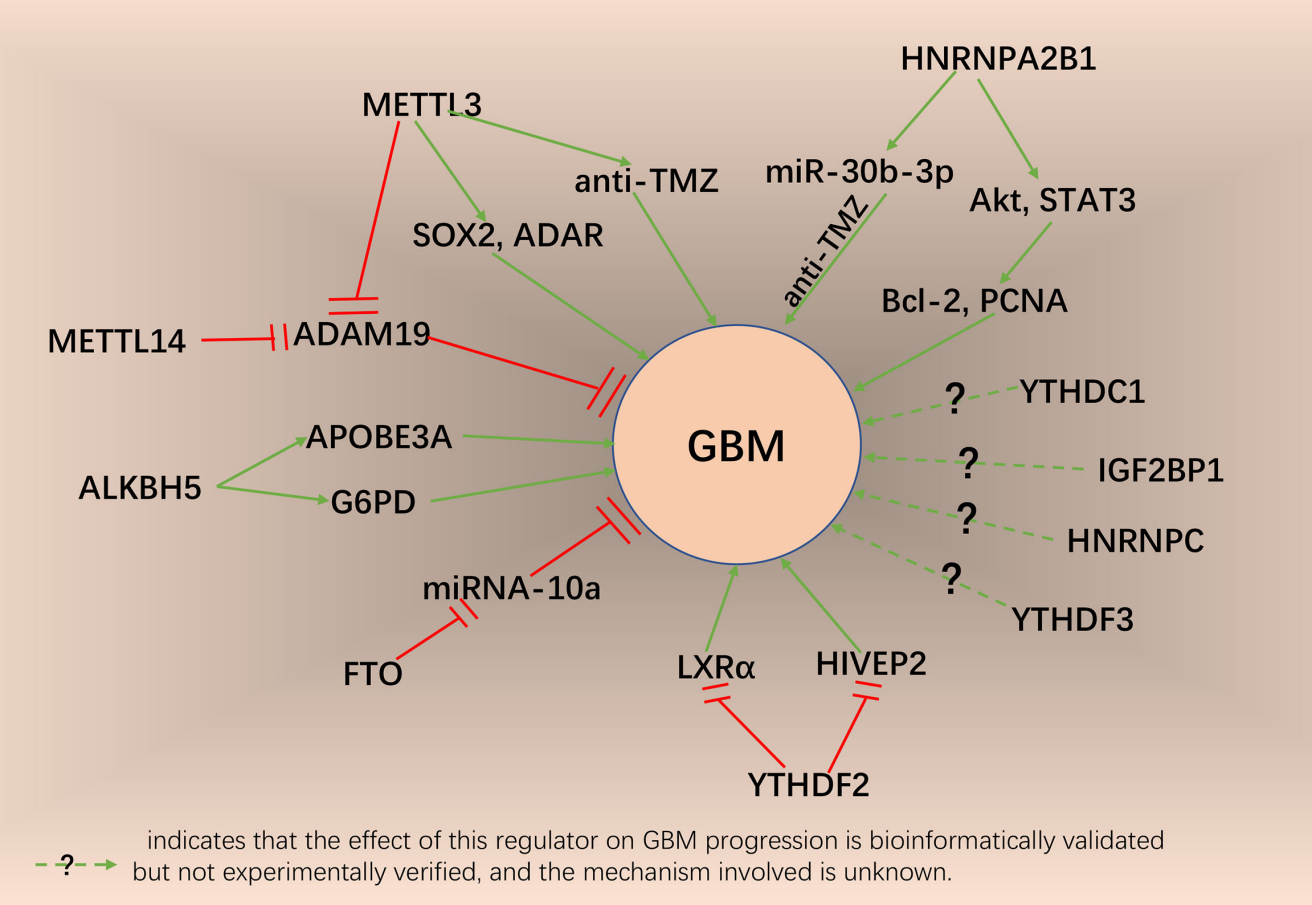
The potential role of m6A regulators in GBM progression and related mechanisms.

Our previous work demonstrated the mechanism of m6A methylation modification related to the regulation of TME immune cell infiltration, stemness and biological processes in GBM (6). We found that the copy number variations status of the four m6A regulators YTHDC1, ALKBH5, FTO, and METTL3 were correlated with the development of GBM (6). Zhu et al. demonstrated that YTHDC1 inhibits glioma cells proliferation by decreasing VPS25 expression (141). Liu et al. demonstrated that ALKBH5 demethylates the target transcript G6PD and enhances its mRNA stability, promotes G6PD translation and activates the pentose phosphate pathway, which in turn promotes glioma cell proliferation (142). Zhang et al. confirmed that FTO can inhibit the proliferation and invasion of GBM cells *in vitro* and *in vivo* by regulating the m6A modification of primary microRNA-10a (143). Shi et al. showed that METTL3-mediated m6A modification was elevated significantly in TMZ-resistant GBM cells, and METTL3 functions as a key promoter of TMZ resistance in GBM, its overexpression impaired the sensitivity of GBM cells to TMZ (144). TMZ induced SOX4-mediated increases in chromatin accessibility at the METTL3 locus, and METTL3 deletion influenced the deposition of m6A on the histone modification-related gene EZH2, leading to nonsense-mediated mRNA decline, so that METTL3 silencing inhibited TMZ-resistant xenograft growth in a synergistic manner when used in combination with TMZ (145). Somatic mutation analysis revealed that more than 10% of GBM patients experienced alterations in m6A regulators, mainly including profound deletions, amplifications and missense mutations, with the highest frequency of mutations in IGF2BP1 (6). Fang et al. demonstrated that YTHDF2 affects the survival of GBM patients by promoting m6A-dependent mRNA decay of LXRα and HIVEP2, and that YTHDF2 promotes tumorigenesis of GBM by downregulating LXRα and HIVEP2 (146). NF-κB activating protein (NKAP) affected GBM progression by binding to m6A to promote the splicing and maturation of SLC7A11 mRNA as a novel ferroptosis inhibitor (147). Our previous study found four m6A regulators, HNRNPC, HNRNPA2B1, ALKBH5, and YTHDF3, to be significantly associated with overall survival by Cox proportional hazards regression analysis of GBM samples in TCGA and CGGA (6). Yin et al. demonstrated that HNRNPA2B1 can mediate the packaging of miR-30b-3p into extracellular vesicles and promotes the ability of GBM cells to resist TMZ (148). It was also shown that knockdown of HNRNPA2B1 in GBM cells could lead to the inactivation of AKT and STAT3 signaling pathways in tumor cells, reduce the expression of Bcl-2 and PCNA, and thus inhibit the growth of GBM cells, and the establishment of xenograft tumor models using GBM cells with knockdown of HNRNPA2B1 also revealed that knockdown of HNRNPA2B1 could inhibit the progression of GBM *in vivo (* 149). Analysis of GBM samples with different m6A scores using K-M analysis showed that overall survival was significantly higher in the group with low m6AScore than in the group with high m6AScore, while the P-value for m6AScore was lower than 0.05 (HR>1) in both uni- and mul-tivariate Cox analysis. Survival of GBM patients between low and high m6AScore groups of different molecular subtypes was assessed using K-M analysis, which showed that the IDH-WT-m6AScore low group had the best survival advantage and the IDH-WT-m6AScore high group had the worst overall survival; among different methylation statuses, the MGMT-methylated-m6AScore low group had the highest overall survival and the MGMT-unmethylated-m6AScore high group had the worst overall survival; the m6AScore low group also had a significant survival advantage over the high group in different x1p19q code statuses (6). **Figure 4** summarized the mechanism by which m6A mediators affect GBM progression.

GBM is a commonly fatal cancer and contains GBM stem cells (GSC) that initiate tumor self-renewal, and GSCs are considered to be a new therapeutic target for GBM (150). The study confirmed that METTL3 facilitates mRNA methylation, increases the stability of SRY transcription factor 2 (SOX2), improves SOX2 protein expression, and contributes to the radiation resistance and maintenance of glioma stem cell-like cells (151). It was shown that downregulation of METTL3 expression decreases the level of m6A modification of serine- and arginine-rich splicing factor (SRSF), which leads to YTHDC1-dependent NMD of SRSF transcripts and reduces expression of SRSF protein, and silencing of METTL3 or overexpression of dominant-negative mutant METTL3 inhibits GSC growth and self-renewal (152). In GSCs, the m6A reader YTHDF2 was highly expressed, while YTHDF2 stabilized MYC and VEGFA transcripts in GSCs in an m6A-dependent manner, exhibiting a role in linking GSC growth and RNA epitranscriptomic modifications (36). ALKBH5 was shown to be highly expressed in GSC, demethylation of FOXM1 nascent transcripts by ALKBH5 leads to enhanced the expression of FOXM1, long non-coding RNA antisense of FOXM1 (FOXM1-AS) facilitates the association of FOXM1 nascent transcripts with ALKBH5, and depletion of ALKBH5 and FOXM1-AS blocks GSC tumorigenesis *via* the FOXM1 axis (153). Our previous study confirmed the important role of m6A modifications in GSC by comparing GBM samples with different m6A modification patterns (6).

## The Immunomodulatory Potential of m6A Modification in GBM

Presently a number of studies have reported a relationship between m6A methylation and immune cell infiltration in the TME (154), but this phenomenon cannot be explained by classical RNA degradation. It has been reported that METTL3-mediated mRNA m6A methylation promotes activation and function of dendritic cells, and knockout of METTL3 in dendritic cells leads to impaired dendritic cell phenotype and functional maturation, decreased expression of co-stimulatory molecules CD40, CD80 and cytokine IL-12, and decreased ability to stimulate T cell responses (155). Yin et al. found that ablation of METTL3 in myeloid cells promoted tumor growth and metastasis, and that METTL3-deficient mice exhibited increased infiltration of M1/M2-like tumor-associated macrophages and regulatory T cells into tumors compared to wild-type mice (156). Dong et al. demonstrated that knockdown of METTL14 in tumor-associated macrophages drives CD8+ T cell differentiation along a dysfunctional trajectory that impairs CD8+ T cells to eliminate tumors, and METTL14-deficient C1q+ tumor-associated macrophages display reduced m6A abundance of the cytokine subunit Ebi3 and elevated levels of transcripts (157). In a model of neuroinflammation, knockdown of ALKBH5 increased m6A modifications of interferon-γ and C-X-C motif chemokine ligand 2 mRNA, thereby reducing their protein expression and the stability of mRNA in CD4+ T cells, and these modifications contributed to an attenuated CD4+ T cell responses and reduced neutrophil recruitment into the CNS (158).

In our previous study, we divided GBM samples into high and low m6AScore groups based on their m6A methylation status and further analyzed the immune cell infiltration of the TME in both groups to identify two clusters of immune phenotypes: immune activation differentiation phenotype and immune desert dedifferentiation phenotype (6). Selective depletion of m6A regulators in tumor-associated macrophages has been demonstrated to reduce infiltration of immunosuppressive cells, thereby benefiting patients receiving immunotherapy (159). In our previous study, we found that inconsistent ratios of pro- and anti-tumor immune cells in TME of single tumor, disruption of oncogenic dedifferentiation phenotypes in different pathways, and dysregulation of distinct signaling pathways may be correlated with the patterns of m6A modification, and that differences in mRNA transcriptomes between distinct m6A modification patterns were strongly associated with immune-related biological pathways (6). In the GBM group with high m6A scores, an immune tolerance phenotype characterized by mesenchymal tissue subtypes and IDH1 wild molecule subtypes, as well as high infiltration of immune cells and stromal cells was demonstrated (160). In the high m6A scoring group despite higher immune checkpoint expression, GBM individuals responded poorly to anti-CTLA4 immunotherapy regimens due to dysfunctional T cells, whereas the low m6A scoring group had an immunodeficient phenotype with less immune cell infiltration and a better prognosis (160). Meanwhile GBM patients in the low m6A score group had higher t-cell exclusion scores and microsatellite instability, as well as better response to anti-CTLA4 immunotherapy (160). Pan et al. demonstrated that m6A modification patterns are closely associated with immune responses, such as neutrophil-mediated immunity and neutrophil activation involved in immune responses (13). In our previous study, we divided the GBM cohort samples into two clusters based on m6A modification patterns and compared the distribution of immune cells in the two clusters and found that: tumor-promoting immune cells (pDC, Neutrophil, CD56dimNK, imDC, Th2, MDSC, TAM, and Treg) were enriched in the poor survival cluster and anti-tumor immune cells (NKT, TemCD4, TemCD8, ActCD4, ActCD8, Th1, ActDC, TcmCD4, TcmCD8, CD56briNK Th17, and NK) were enriched in the cluster with a survival advantage (6). Pan et al. demonstrated that in the GBM microenvironment, the expression level of the m6A regulator ELAVL1 was negatively associated with the infiltration of most immune cells, except for activated CD4+ T cells and type 2 helper T cells (13). Knockdown of ALKBH5 in GBM cells significantly inhibited hypoxia-induced recruitment and immunosuppression of tumor-associated macrophages in allograft tumors, and CXCL8/IL8 expression and secretion were significantly suppressed (161). Hypoxia-induced ALKBH5 in GBM cells cleared m6A deposition of lncRNA NEAT1, stabilized transcripts and facilitated NEAT1-mediated parabasal assembly, which led to relocalization of the transcriptional repressor SFPQ from the promoter of CXCL8 to the parabasal and thereby promoted the expression of CXCL8/IL8 (161). Qi et al. found that miR-454-3p inhibits m6A modification by binding to YTHDF2 enzyme, and histone methyltransferase EZH2 inhibits miR-454-3p by methylation modification and facilitates m6A modification of PTEN to increase M2 macrophage polarization in glioma cells (162). JMJD1C is a H3K9 demethylase and miR-302a can target METTL3, which can inhibit SOCS2 expression through m6A modification. Zhong et al. demonstrated that JMJD1C facilitates macrophage M1 polarization in the glioma microenvironment through the miR-302a/METTL3/SOCS2 axis *in vivo* vitro and inhibits tumor growth (163). Pan et al. found that in glioma cells, HNRNPA2B1 could contribute to the packaging of circNEIL3 into exosomes and delivery to infiltrating tumor-associated macrophages in TME, allowing them to acquire immunosuppressive characteristics by stabilizing IGF2BP3, which in turn facilitates the progression of glioma (164). YTHDC2 has also been shown to play an important role in the immune infiltration of the microenvironment of low-grade glioma and is a potential biomarker for its diagnosis and prognosis (165).

## Novel Immunotherapy in GBM and Other Cancers

A growing and promising field of novel immunotherapy is represented by anti-PD-1/PD-L1 (immune checkpoint blockade). Our previous study observed that the relationship between anti-PD-L1 and anti-PD-1 treatment response and m6A modification patterns was consistent with the relationship between GBM and m6A modification patterns (6), which we confirmed by establishing the m6AScore system (6), and our analysis suggests that it may be due to the relatively high component of immune cell infiltration in the high m6AScore group. It was found that neoadjuvant PD-1 blockade in GBM cells increased the proportion of T cell infiltration and progenitor-depleted T cell populations found within tumors (166). The authors also identified an early activated and clonally expanded cluster of CD8+ T cells with a TCR that overlapped a population of CD8+ PBMC, and significant changes were also noted in type 1 dendritic cells, which may promote T cell recruitment. Moreover, monocytes and macrophages remain a major component of infiltrating immune cells even after anti-PD-1 treatment (166). In our previous study analyzing the m6A methylation status of the anti-PD-1 treated GBM cohort described above, we observed that almost all anti-tumor immune cells were enriched in the low m6A scoring group and almost all pro-tumor immune cells were enriched in the high m6A scoring group; most of the classical oncogenic pathways were enriched in the high m6A scoring group (6). The YY1-CDK9 complex is pharmacologically or genetically targeted to induce RNA m6A modification-dependent interferon responses, decrease the infiltration of regulatory T cells, and enhance the efficacy of GBM immune checkpoint therapy (167). In GC, the low m6A signaling group showed a higher neoantigen load and elevated anti-PD-1/PD-L1 immunotherapy response, and two immunotherapy cohorts in melanoma and urothelial carcinoma confirmed that patients with lower m6A signaling showed significant benefits of treatment and were clinically advantageous (74). The uroepithelial cancer cohort was grouped by ELAVL1 expression and the proportion of patients responding to PD-L1 blockade immunotherapy in the low ELAVL1 or high ELAVL1 expression groups was analyzed, indicating that ELAVL1 high expression was associated with a relatively effective response to PD-L1 therapy (13). *In vitro* experiments, YTHDF1 and YTHDF2 knockdown in NSCLC cells upregulated tumor PD-L1 expression and changed a variety of immune-related genes, while high expression of YTHDF1 and YTHDF2 was associated with good prognosis, a large number of tumor-infiltrating lymphocytes, and downregulation of PD-L1 in NSCLC patients (168). In the NSCLC microenvironment, METTL3 could mediate the m6A modification of circIGF2BP3 and facilitate its recycling in a YTHDC1-dependent manner. circIGF2BP3 reduces PD-L1 ubiquitination and subsequent proteasomal degradation by enhancing OTUB1 mRNA stability in a PKP3-dependent manner, leading to immune evasion of CD8+ T cell-mediated killing (169). It was found that there were significant differences in overall survival and immune cells infiltration between different m6A subgroups of cutaneous melanoma (170). The m6A score was positively associated with regulatory T-cell and helper T-cell content, which may explain why a high m6A score is associated with a better prognosis (170). Furthermore, high m6A score patients presented a stronger response to novel immunotherapies, and two immune-related samples receiving anti-PD-1 or anti-PD-L1 therapy confirmed that patients in the high m6A score group had a better response to novel immunotherapy (170).

## Summary and Conclusions

In our present review, we comprehensively summarized the pathophysiological functions of m6A regulators, the modification patterns of m6A, the potential role of m6A in cancer and CSCs, the role of m6A modifications on the tumor immune microenvironment, and the role of m6A modifications in novel immunotherapy of tumors, especially in GBM. The m6A modification process occurs primarily in the adenine of the RRACH sequence, a dynamic reversible biological process in which writers catalyze the installation of m6A on RNA and erasers remove the modifications. The reader’s recognition of m6A methylation influences mRNA splicing, export, degradation, and translation, modifies the underlying biological processes accordingly. m6A and its associated regulators play multiple significant roles in cancers, mechanistically because m6A methylation and its associated regulators can influence the processing of miRNAs and the biological functions of lncRNAs as well as can facilitate circRNAs’ translation.

Importantly, the effect of m6A on cancer progression appears to be bidirectional. Some genes can facilitate tumor progression after methylation, while others can inhibit tumor progression after methylation. This is also highlighted by the effect of m6A on TME immune cell infiltration. In GBM, m6A methylation modification facilitated the enrichment of pro-tumor immune cells and promoted tumor development. In contrast, in gastric cancer, m6A methylation modification appeared to show a bidirectional effect on TME immune cell infiltration, with both pro-tumor and anti-tumor immune cells enriched in the high m6A methylation modification group. Anti-PD-1/PD-L1 is a growing and promising area for novel immunotherapy. The role of m6A methylation modifications in novel immunotherapy has been demonstrated, but the limited research is currently focused on peripheral cancers. About m6A methylation modifications in novel immunotherapies for GBM is still lacking in detail.

The development of epitranscriptomics has provided new explanations for the discovery of biological mechanisms of cancer development, and m6A modification is the most representative of them, providing new targets for cancer treatment. However, the current understanding of m6A modifications especially in GBM is still limited, and we believe that upcoming studies on m6A modifications will focus on the following four points: first, quantifying m6A methylation modifications in individual tumors with possible future application as new biomarkers for predicting cancer recurrence, selecting therapies, and identifying treatment response; second, to investigate the relationship between novel immunotherapies and m6A modifications in GBM, the effect of m6A modifications on macrophage polarization in the GBM microenvironment and its mechanisms; third, the effect of m6A modification on the biological behavior of GBM stem cells and its role in the maintenance of GBM cell stemness: fourth, to identify m6A-related antigens and immune subtypes in GBM for the development of mRNA vaccines.

## Author Contributions

FC wrote the original draft and contributed to drawing. XX, MC, HC revised the manuscripts. LW provided ideas. All authors contributed to the article and approved the submitted version.

## Funding

This study was supported by the Fund of Tang Du hospital (No.2021YFJH005, No. 2021SHRC033).

## Conflict of Interest

The authors declare that the research was conducted in the absence of any commercial or financial relationships that could be construed as a potential conflict of interest.

## Publisher’s Note

All claims expressed in this article are solely those of the authors and do not necessarily represent those of their affiliated organizations, or those of the publisher, the editors and the reviewers. Any product that may be evaluated in this article, or claim that may be made by its manufacturer, is not guaranteed or endorsed by the publisher.
